# Pager-gestütztes Wartezeitmanagement in einer universitären HNO-Ambulanz: Pilotprojekt für mehr Abstand und für mehr Komfort

**DOI:** 10.1007/s00106-021-01023-2

**Published:** 2021-03-16

**Authors:** V. Vielsmeier, A. Brosig, A. Hauser, C. Bohr

**Affiliations:** 1 Klinik und Poliklinik für Hals-Nasen-Ohrenheilkunde, Universitätsklinikum Regensburg, Franz-Josef-Strauss-Allee 11, 93053 Regensburg, Deutschland; 2 Abteilung für Patientenmanagement und Erlössicherung, Universitätsklinikum Regensburg, Regensburg, Deutschland

**Keywords:** COVID-19, Hygiene, Abstandsregeln, Pager, Wartezeitenmanagement, COVID-19, Hygiene, Distancing rules, Pager, Waiting time management

## Abstract

**Hintergrund:**

Aufgrund der Pandemie mit dem neuartigen Coronavirus (SARS-CoV-2) sollte überall auf ausreichend Abstand zwischen Personen geachtet werden, insbesondere auch in Wartebereichen medizinischer Versorgungseinrichtungen. Bei oftmals eingeschränkten räumlichen Kapazitäten ist dies jedoch nicht immer problemlos realisierbar.

**Ziel der Arbeit:**

Wir untersuchten die Möglichkeit, mittels eines Pagersystems den Patienten unserer HNO-Ambulanz eine Wartezeit außerhalb des eigentlichen Wartebereichs zu ermöglichen, um damit die Anzahl der Patienten im Wartebereich zu reduzieren, und die Einhaltung der Abstandsregeln zu gewährleisten.

**Material und Methoden:**

In einer Zeitspanne von 12,5 Wochen (Beginn 04.06.2020, Ende 31.08.2020) erfolgte die Ausgabe von Pagern an die Patienten unserer HNO-Ambulanz. Teilnehmenden Patienten war es damit möglich, sich während der Wartezeit auf dem gesamten Klinikgelände frei zu bewegen. Der Pager wurde 10–15 min vor dem Termin aktiviert, und der Patient damit zurück in die HNO-Ambulanz gerufen. Mittels Fragebögen erfolgte eine Evaluation des Systems, um die Akzeptanz und Zufriedenheit der Patienten zu eruieren.

**Ergebnisse:**

137 Fragebögen wurden analysiert, hierbei zeigte sich eine Zufriedenheit mit dem System – nicht nur, was die Einhaltung der Abstandsregeln betrifft, sondern auch bzgl. eines höheren Komforts während der Wartezeit.

**Schlussfolgerung:**

Die Einführung eines Pagersystems für Patienten führt neben der Wahrung der Hygiene- und Abstandsregeln auch zu einer Erhöhung des Komforts während der (häufig nicht zu vermeidenden) Wartezeit für die Patienten in der universitären Ambulanz einer HNO-Klinik. Daher erscheint ein langfristiger Einsatz eines solchen Systems vielversprechend.

## Abstract (en)

**Background:**

Due to the severe acute respiratory syndrome coronavirus 2 (SARS-CoV-2) pandemic, sufficient distance between people is absolutely necessary, especially in waiting rooms of medical care institutions. This is frequently difficult to realize due to restricted spatial capacities.

**Objective:**

This study investigated the possibility of enabling patients to spend the waiting period outside of the waiting room of the ENT outpatient department, in order to reduce the number of patients in the waiting rooms and therefore observe distancing rules. This was realized by the use of a pager system.

**Materials and methods:**

During the timeframe of 12.5 weeks (04.06.2020–31.08.2020), ENT outpatients were issued with pagers. The patients could thus move freely within the hospital and grounds. The pager system was activated 10–15 min before the appointment, to call patients back to the outpatient ENT clinic. questionnaires were used to evaluate the system and examine patients’ acceptance and satisfaction.

**Results:**

The 137 questionnaires analyzed showed satisfaction with the system, not only regarding distancing rules but also with the more comfortable waiting time.

**Conclusion:**

Introduction of a pager system for patients could help to meet hygiene and distance rules, and also increase comfort during (often unavoidable) waiting times for patients in the university hospital ENT outpatient department. The long-term use of such a system seems promising.

Die Einhaltung der Hygiene- und Abstandsregeln im Rahmen der COVID-19-Pandemie erfordert auch in Wartebereichen von medizinischen Einrichtungen entsprechende Maßnahmen. Wir stellen ein Pilotprojekt in unserer HNO-Klinik vor. Wir etablierten ein Pagersystem, durch das einerseits Abstände zwischen den Patienten besser eingehalten werden konnten und andererseits der Komfort während der Wartezeit erhöht werden konnte.

## Mindestabstände in einer universitären Ambulanz in Zeiten einer Pandemie

Aufgrund der Corona-Pandemie wurden die bayerischen Krankenhäuser, darunter auch die Universitätsklinika, per Allgemeinverfügung vom 19. März 2020^1^ aufgefordert, alle planbaren Behandlungen – soweit medizinisch vertretbar – zurückzustellen oder zu unterbrechen. Gründe hierfür waren die Schonung logistischer und personeller Ressourcen, die Unterbrechung von Infektionsketten sowie die Vorhaltung von Kapazitäten in den Kliniken für die Versorgung von SARS-CoV-2-Patienten. Auch der Ambulanzbetrieb in den Kliniken wurde in dieser Zeit auf die Notfallversorgung beschränkt.

Eine Analyse der Patientenzahlen für diesen Zeitraum des Notfallbetriebs ergab deutlich reduzierte Patientenzahlen im Vergleich zum gleichen Zeitraum des Vorjahrs. In dieser Zeit konnte der Ambulanzbetrieb bei übersichtlichen Patientenzahlen daher trotz der einzuhaltenden Hygiene- und Abstandsregeln problemlos aufrechterhalten werden.

Jedoch zeigte sich bei zunehmender Rückkehr zum Normalbetrieb, dass die steigende Anzahl der Patienten nicht mehr ohne Weiteres im Wartebereich aufgenommen werden konnte.

Es mussten Maßnahmen ergriffen werden, um die Patientenströme unter Berücksichtigung der Erfordernisse einer pandemischen Situation bewältigen zu können [2]. Problematisch war dabei vor allem, den Abstand in den Wartebereichen bei vorhandenem Platzmangel einzuhalten. Ein Abstand von mindestens 1,5 m, zum Teil sogar mindestens 2 m gilt jedoch neben anderen Hygienevorschriften als essenzielle Maßnahme in der Prävention von Infektionsketten [1, 4]. Bereits mit der ersten Bayerischen Infektionsschutzmaßnahmenverordnung (BayIfSMV) vom 27.03.2020^2^ wurde ein Abstand von mind. 1,5 m für diverse Bereiche des Alltagslebens vorgeschrieben. Mit der vierten BayIfSMV vom 05.05.2020 folgte dann ein allgemeines Abstandsgebot von mindestens 1,5 m zwischen 2 Personen für alle Bereiche des öffentlichen Lebens.

Durch Notfälle und häufig unerwartet aufwendigere Versorgung der Patienten sind längere Wartezeiten in einer universitären Ambulanz leider häufig nicht zu vermeiden. Allerdings ist eine Korrelation von Wartezeit und Unzufriedenheit der Patienten bekannt [10]. Auch wenn eine Reduktion der Wartezeit eine große Herausforderung darstellt, könnten strukturelle und organisatorische Maßnahmen sowie die Verbesserung der Wartezeit die Zufriedenheit der Patienten in einer Ambulanz erhöhen [3, 10].

In unserer HNO-Ambulanz wurde daher ein Pagersystem etabliert, mit dessen Hilfe die Patienten sich während der Wartezeit in der gesamten Universitätsklinik und dem dazugehörigen Gelände aufhalten konnten. Kurz vor ihrem Termin in der Ambulanz wurden sie mithilfe des Pagers angefunkt, um dann zur Poliklinik zurückzukehren. Damit konnten mehr Patienten versorgt werden, ohne dass die Abstands- und Hygieneregeln verletzt wurden.

## Methoden

In unserer HNO-Ambulanz mussten im Zeitraum von 04.06.2020 bis 31.08.2020 insgesamt 4343 Patienten an 89 Wochentagen versorgt werden. Gleichzeitig mussten pandemiebedingt zur Einhaltung der Abstandsregeln einige Sitzplätze im Wartebereich gesperrt werden. Um das Patientenaufkommen dennoch bewältigen zu können, etablierten wir ein System mit Pagern, durch das die Patienten den Wartebereich während der Wartezeit verlassen konnten. Zur Veranschaulichung des Patientenaufkommens und der Wartesituation in unserer HNO-Ambulanz wurden zudem Daten zu Patientenbewegungen und Wartebereich aufgelistet.

## Ablauf der Benutzung des Pagersystems

Als Pager wurde das alte funkbasierte Kommunikationssystem des Universitätsklinikums Regensburg genutzt, das 2018 durch DECT(„digital enhanced cordless telecommunication“)-Telefone ersetzt wurde. Nachdem die Funkempfänger seither nicht mehr in Benutzung sind, konnten 20 Stück für das Pilotprojekt zum Pager-gestützten Wartezeitenmanagement in unserer HNO-Ambulanz verwendet werden. Sobald sich der Wartebereich der HNO-Ambulanz mit Patienten füllte bzw. die Patientenzahlen anstiegen, wurden die Pager an die Patienten ausgegeben. Die Verwendung der Pager war dabei freiwillig. Der Funkempfänger des betreffenden Patienten wurde 10–15 min vor dem Termin aktiviert (Ertönen eines lauten Tonsignals), und der Patient damit zur Leitstelle der HNO-Ambulanz gerufen. Die Patienten mussten dann noch einige Minuten im Wartebereich verbleiben, bis sie von dort zur ärztlichen Behandlung gerufen wurden. Wir analysierten die Patientenbewegungen und die zur Verfügung stehenden Flächen in unserem Wartebereich. An die teilnehmenden Patienten wurde ein selbst entworfener zweiseitiger Fragebogen ausgegeben, in dem die Zufriedenheit und die Erfahrungen mit diesem System evaluiert wurden.

**Figure 1:**
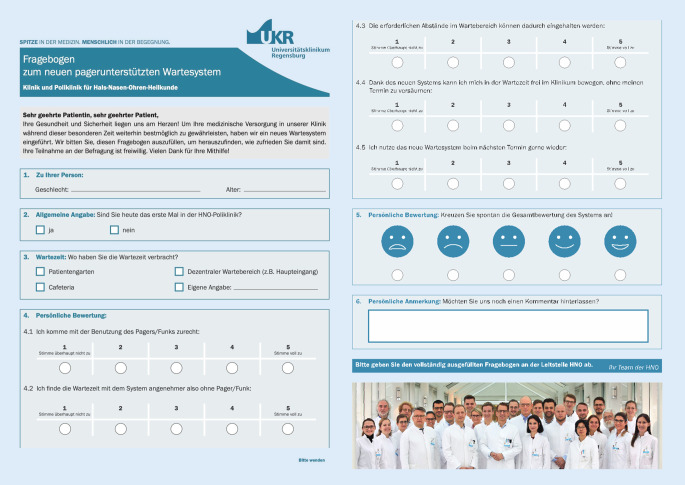

Hierbei wurde nach Alter und Geschlecht des Patienten gefragt, außerdem erfolgte eine Bewertung des Pagersystems mittels verschiedener Fragen und visueller Analogskalen. Darüber hinaus wurde eruiert, in welchen Bereichen des Klinikums die Patienten die Wartezeit verbrachten. Der vollständige Fragebogen ist in Abb. 1 abgebildet.

## Patientenbewegungen in der HNO-Ambulanz

Im Testzeitraum (zwischen 04.06.2020 und 31.08.2020) wies unsere HNO-Ambulanz insgesamt 6254 Patientenbewegungen auf. Im Vergleich dazu waren es im gleichen Zeitraum des Vorjahrs 6923 Bewegungen, das entspricht einer Abnahme von nur 9,66 % und zeigt den starken Nachholeffekt nach den zeitweisen Beschränkungen unserer Ambulanz auf den Notfallbetrieb. Ein Patient weist dabei mehrere Bewegungen am selben Tag auf, wenn er an einem Tag mehrere Termine in unterschiedlichen Bereichen unserer HNO-Ambulanz absolvierte (z. B. Termin in der klassischen HNO-Ambulanz und in der Phoniatrie). Betrachtet man die Patientenbewegungen an den Wochentagen (Montag bis Freitag), so fanden insgesamt 6201 Bewegungen an 63 Tagen statt, das entspricht einer durchschnittlichen Anzahl von 98,43 Bewegungen an einem Wochentag in unserer Ambulanz.

## Wartebereiche in der HNO-Ambulanz

Der Wartebereich unserer HNO-Ambulanz hat eine Fläche von 57 m^2^, die Anzahl der Sitzplätze liegt regulär bei 40.

Bei einem geforderten Mindestabstand von 1,5 m zwischen den Patienten wäre pro Patient eine freie Fläche von 1,77 m^2^ erforderlich. Die Anzahl an Sitz- und Stehplätzen im Wartebereich berücksichtigend, wäre damit theoretisch der Aufenthalt von 32 Personen gleichzeitig möglich. Durch die eingeschränkte Anzahl an Sitzplätzen im Wartebereich waren unter Berücksichtigung der Hygieneregeln allerdings nur 18 Patienten gleichzeitig erlaubt. Problematisch wirkt hierbei noch, dass in einer universitären Ambulanz die Aufenthaltszeit der Patienten aufgrund der Komplexität der Fälle mit mehreren notwendigen Untersuchungen und Konsilen, der Wartezeit auf einen Oberarzt und der variablen personellen Besetzung sehr unterschiedlich sein kann. Eine berechnete durchschnittliche gesamte Aufenthaltsdauer in der Ambulanz von 1 h und 23 min, welche sich aus 100 Patientenbewegungen pro durchschnittlichem Wochenarbeitstag mit einer Dauer von 7,6 h bei 18 vorhandenen Sitzplätzen errechnet, erscheint zunächst ausreichend Zeit zu sein. Aus der praktischen Erfahrung wird jedoch klar, dass einige Patienten diese Zeitspanne deutlich überschreiten und damit den Wartebereich über einen noch längeren Zeitraum nicht verlassen können.

Hierbei ist außerdem auch zu beachten, dass ein Teil der Patienten mindestens einen Angehörigen oder eine Begleitperson mitbringt, welcher zum Patienten selbst den Mindestabstand als direkte Kontaktperson zwar nicht einhalten muss, aber dennoch Platz im Wartebereich einnimmt.

## Ergebnisse

Die Fragebögen von 137 Patienten wurden analysiert und mittels Excelprogramm berechnet. Die Aufteilung zwischen den Geschlechtern lag bei 81 (59,1 %) männlichen und 53 (38,7 %) weiblichen Personen, 3 Personen (2,2 %) haben keine Angabe zum Geschlecht gemacht. Das Durchschnittsalter lag bei 48,6 Jahren. 43,1 % der Patienten waren zum ersten Mal in unserer HNO-Ambulanz vorstellig, 56,9 % waren dagegen bereits vorher einmal Patient in unserer Ambulanz. Die teilnehmenden Patienten gaben an, die Wartezeit zu 56,9 % im Garten, zu 33,6 % dezentral und zu 10,2 % im Klinikcafé verbracht zu haben, wobei von einzelnen Personen auch mehrere Wartebereiche während der Wartezeit genutzt wurden.

**Figure 2:**
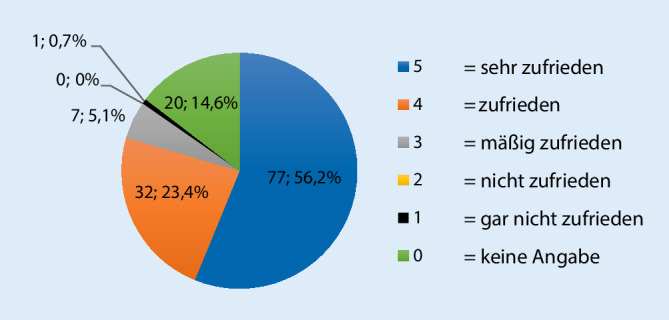

In den Fragebögen wurden hierbei die Themen Empfinden der Wartezeit, Einhaltung der Abstände, Terminversäumnis und Befürwortung einer Wiederverwendung des Systems beim nächsten Termin abgefragt. Die Gesamtbewertung ist in Abb. 2 zu erkennen. Hierbei bedeutete eine Vergabe von 5 Punkten, dass die Patienten „sehr zufrieden“ mit dem Pagersystem waren, während 1 Punkt „gar nicht zufrieden“ repräsentierte. Insgesamt wurden 5 Punkte für die Gesamtzufriedenheit von 56,2 % der Nutzer vergeben, während nur eine von 137 Personen gar nicht zufrieden war. Weitere 23,4 % waren zufrieden mit den Pagern.

Der Mittelwert der Gesamtzufriedenheit betrug 4,6 von 5 Punkten. Beim Großteil der Patienten war auch die Zufriedenheit mit der Benutzungserfahrung (112 Patienten, 81,8 %), dem Empfinden einer angenehmeren Wartezeit (85; 62,0 %), dem Abstandseinhalten (82; 59,9 %), dem fehlenden Terminversäumnis (97; 70,8 %) und auch der Wunsch zur erneuten Benutzung eines Pagersystems (96, 70,1 %) sehr hoch (Tab. 1).

**Table 1:** 

Bewertung	Benutzungserfahrung (*n*)	Angenehmere Wartezeit (*n*)	Abstandseinhalten (*n*)	Kein Terminversäumnis (*n*)	Wunsch zur erneuten Benutzung (*n*)	Gesamt (*n*)
5	112 (81,8 %)	85 (62,0 %)	82 (59,9 %)	97 (70,8 %)	96 (70,1 %)	*77 (56,2* *%)*
4	9 (6,6 %)	14 (10,2 %)	20 (14,6 %)	13 (9,5 %)	9 (6,6 %)	*32 (23,4* *%)*
3	9 (6,6 %)	16 (11,7 %)	10 (7,3 %)	3 (2,2 %)	7 (5,1 %)	*7 (5,1* *%)*
2	2 (1,5 %)	5 (3,6 %)	3 (2,2 %)	2 (1,5 %)	2 (1,5 %)	*0*
1	2 (1,5 %)	12 (8,8 %)	0	2 (1,5 %)	3 (2,2 %)	*1 (0,7* *%)*
Ka. (keine Angabe) (= 0)	3 (2,1 %)	5 (3,6 %)	21 (15,3 %)	20 (14,6 %)	20 (14,6 %)	*20 (14,6* *%)*
Mittelwert	4,7	4,2	4,6	4,7	4,6	4,6
Median	5	5	5	5	5	5

In den Freitextergebnissen wurde angemerkt, dass dadurch der Service erhöht wurde, die Wartezeit „erholsamer“, „angenehmer“ und/oder „stressfreier“ war.

Zwischen den Bewertungen durch Männer und Frauen gab es keinen signifikanten Unterschied, jedoch zeigten sich Auffälligkeiten beim Vergleich verschiedener Altersgruppen. Der Mittelwert der durchschnittlichen Gesamtbewertung einzelner Altersgruppen liegt bei 4,57. Die durchschnittliche Gesamtbewertung ist umso schlechter, je älter die Patienten sind (Altersgruppe unter 30 Jahre: Mittelwert 4,65; zwischen 65 und 70 Jahre: Mittelwert 4,33). Somit ist eine Tendenz zu erkennen, dass jüngere Patienten mit dem Pagersystem insgesamt zufriedener sind als ältere Patienten.

## Diskussion – Vor- und Nachteile des Pagersystems

Die COVID-19-Pandemie führt zu einer erforderlichen Neuorganisation einer HNO-Klinik, hierzu gehören unter anderem das konsequente Tragen von Schutzausrüstungen, aber auch diverse organisatorische Maßnahmen wie das Verschieben elektiver Eingriffe oder die Reduktion von Besuchern und Begleitpersonen [7].

Darüber hinaus sind im Speziellen diverse Aspekte im Management einer Ambulanz zu beachten, hierzu gehören neben den üblichen Schutz- und Hygienemaßnahmen die Reduktion der Termine und damit die Vermeidung von größeren Ansammlungen im Wartebereich [8].

Jedoch sind diese Maßnahmen häufig nicht ausreichend, um einen ausreichenden Abstand von mindestens 1,5 m zwischen Personen im Wartebereich zu gewährleisten. Daher führten wir das hier beschriebene Pilotprojekt mit einem Pagersystem durch. Primäres Ziel der Einführung des Pagersystems für die Patienten im Wartebereich der HNO-Ambulanz war die Vermeidung von engen Kontakten, die Einhaltung der Abstandsregeln und damit die Wahrung der Hygienevorschriften.

Jedoch zeigten sich darüber hinaus weitere Vorteile dieses neuen Systems. So empfinden die Patienten durch mehr Optionen, die Wartezeit zu verbringen, diese als angenehmer. Auch das Gefühl, dass andere Patienten eventuell bevorzugt oder früher aufgerufen werden, entfällt, da sich die Patienten nicht im Wartebereich befinden und eine subjektiv von den Patienten wahrgenommene Reihenfolge gar nicht erst entstehen kann. In anderen Beobachtungen (z. B. in einer Radio-Onkologie- oder Chemotherapie-Ambulanz) konnten bei insgesamt positiver Bewertung, unter anderem wegen größerer Bewegungsfreiheit, auch die Nachfragen der Patienten beim Klinikpersonal nach Wartezeiten durch ein Pagersystem reduziert werden [5, 6]. In unseren Fragebögen wird darüber hinaus positiv bewertet, dass die Abstände besser eingehalten werden können.

Im Rahmen einer Untersuchung mit Pagern in einer pädiatrischen Notaufnahme zeigte sich jedoch, dass die Zufriedenheit insgesamt durch das System nicht wesentlich erhöht werden konnte [9]. Auch bei uns fiel auf, dass bestimmte Patientengruppen das System weniger positiv evaluierten. So bewerteten Patienten mit höherem Alter das System tendenziell schlechter. Gründe hierfür könnten eine eingeschränkte Mobilität und schlechteres Hörvermögen älterer Patienten sein. Hier könnte mit haptischen oder visuellen Ergänzungen der Pager (Vibrieren und/oder Blinken) eine Optimierung für diese Patientengruppe ermöglicht werden. Dabei ist jedoch zu bedenken, dass ältere Patienten tendenziell nicht so mobil sind und die Möglichkeiten der freien Bewegung in der Klinik bzw. auf dem Klinikgelände nur bedingt oder gar nicht nutzen können. Dies zeigte sich auch darin, dass ältere Patienten das System auf Nachfrage häufiger nicht nutzen wollten und angaben, die Zeit lieber im Wartebereich zu verbringen. Auch eine genaue Definition der Reichweite könnte zur besseren Akzeptanz des Systems führen.

Für unser Pilotprojekt wurde das alte Funksystem des Uniklinikums reaktiviert. Aufgrund der positiven Resonanz, der hohen Praktikabilität und der weiterhin auf hohem Niveau liegenden Infektionszahlen evaluieren wir derzeit die Investition in ein modernes Pagersystem, um in Zukunft die Vorteile und Annehmlichkeiten für die Patienten zu gewährleisten.

## Zusammenfassung – Pagersysteme in Zukunft erforderlich

Die Evaluation des Pagersystems in unserer Ambulanz zeigte durchweg positive Ergebnisse. Dies betrifft einerseits die Einhaltung der Hygieneregeln und andererseits auch die Patientenzufriedenheit, auch wenn dies bestimmte Patientengruppen mehr betrifft als andere. Aufgrund der aktuellen Entwicklung der Infektionszahlen und der weiterhin zwingend erforderlichen Einhaltung der Abstandsregeln ist die Etablierung eines solchen Systems für verschiedene Ambulanzkliniken unabdinglich, um das Patientenaufkommen ohne Verletzung der Hygieneregeln bewältigen zu können.

## Fazit für die Praxis

- Ein Pagersystem im Rahmen des Wartezeitenmanagement wird sehr positiv von den Patienten bewertet.
- Hygiene- und Abstandsregeln in Wartebereichen können dadurch gut eingehalten werden.
- Aufgrund der hohen Infektionszahlen erscheinen derartige Systeme in universitären Ambulanzen in nächster Zeit unabdinglich.

## Footnotes

1 Vollzug des Infektionsschutzgesetzes (IfSG), Corona-Pandemie: Verschiebung elektiver Eingriffe und geplanter Behandlungen in Krankenhäusern, Bekanntmachung des Bayerischen Staatsministeriums für Gesundheit und Pflege vom 19.03.2020, Az. G24-K90000-2020/125; BayMBL 2020 Nr. 151.

2 Bayerische Verordnung über Infektionsschutzmaßnahmen anlässlich der Corona-Pandemie (BayIfSMV) vom 27.03.2020, BayMBl. 2020 Nr. 158.
